# Assessing Skin Cancer Risk Factors, Sun Safety Behaviors and Melanoma Concern in Atlantic Canada: A Comprehensive Survey Study

**DOI:** 10.3390/cancers15153753

**Published:** 2023-07-25

**Authors:** François Lagacé, Bibi Nuzha Noorah, Santina Conte, Lorena Alexandra Mija, Jasmine Chang, Leila Cattelan, Jonathan LeBeau, Joël Claveau, Irina Turchin, Wayne Gulliver, Robert Gniadecki, Elena Netchiporouk, Wilson H. Miller Jr., Thomas G. Salopek, Elham Rahme, Sandra Peláez, Ivan V. Litvinov

**Affiliations:** 1 Division of Dermatology, Faculty of Medicine, McGill University, Montréal, QC H4A 3J1, Canada; francois.lagace@mail.mcgill.ca (F.L.);; 2 Faculté de Médecine, Université de Montréal, Montréal, QC H3T 1J4, Canada; 3 Department of Medicine, Faculty of Medicine and Health Sciences, McGill University, Montréal, QC H4A 3J1, Canada; 4 Department of Family Medicine, McGill University, Montréal, QC H4A 3J1, Canada; 5 Centre Hospitalier Universitaire de Québec, Hôtel-Dieu de Québec, Melanoma and Skin Cancer Clinic, Québec City, QC G1R 4H6, Canada; 6 Division of Dermatology, Dalhousie University, Halifax, NS B3H 2Y9, Canada; 7 Division of Dermatology, Department of Medicine, Memorial University of Newfoundland, St. John’s, NL A1C 5S7, Canada; 8 Division of Dermatology, University of Alberta, Edmonton, AB T6G 2B7, Canada; 9 Department of Medicine and Oncology, McGill University, Montréal, QC H3T 1J4, Canada; 10 Division of Clinical Epidemiology, McGill University, Montréal, QC H3T 1J4, Canada; 11 École de Kinésiologie et des Sciences de L’activité Physique, Université de Montréal, Montréal, QC H3C 3J7, Canada; sandra.pelaez@mail.mcgill.ca

**Keywords:** melanoma, skin cancer, epidemiology, risk factors, UV exposure, sun protection, skin cancer prevention

## Abstract

**Simple Summary:**

Cutaneous melanoma is a deadly form of skin cancer, and its incidence rate continues to increase in Canada. Cutaneous melanoma has many modifiable risk factors, making it a preventable disease. The purpose of this study is to gain valuable information on sun exposure, sun protection and level of worry for cutaneous melanoma in Atlantic Canada. We compare these findings between provinces with a high incidence of cutaneous melanoma against those with a low incidence, as well as between various demographic groups. Our study was able to identify significant differences across multiple variables between these groups. These findings will help guide future public health efforts aiming at preventing cutaneous melanoma and reducing its incidence in our communities.

**Abstract:**

Background: The incidence of cutaneous melanoma (CM) is increasing at an alarming rate in Canada and elsewhere around the world. Significant regional differences in CM incidence have been identified in Atlantic provinces. The goal of this study is to compare ultraviolet exposure, sun protective behaviours, level of worry and baseline CM knowledge in provinces with a high versus low incidence of CM as well, as between various demographic groups. Methods: A cross-sectional survey study was conducted in Atlantic provinces between July 2020 and August 2022. All participants aged ≥ 16 years with a completed survey were eligible. Survey responses were summarized using frequency counts, percentages, and means. Two-sided Z-tests for equality of proportions and logistic regression models were used to compare the survey results between geographic and demographic groups. Results: In total, 7861 participants were included (28.0% men; mean age 61.3 years; response rate 28%). Our results (gender- and age-adjusted odds ratio, 95% confidence interval) show that high-incidence provinces for CM (Prince Edward Island and Nova Scotia) had significantly more sunburns (OR 2.00, 1.72–2.31), total sun exposure (OR 2.05, 1.68–2.50), recreational sun exposure (OR 1.95, 1.61–2.35) and tans (OR 1.77, 1.53–2.05) than individuals in low-incidence provinces (Newfoundland and Labrador). However, individuals in high-incidence provinces displayed more protective behaviors: there were less tanning bed users (OR 0.82, 0.71–0.95), they checked their skin more frequently for new moles (OR 1.26, 1.06–1.51) and practiced more sun protection overall. Additional analyses are presented based on education, income, sexual orientation and gender. Discussion: These findings suggest that future efforts aimed at reducing the CM burden in Atlantic Canada should be tailored for target geographic and/or demographic groups. Limitations: the study participants are not representative of the population in Atlantic Canada due to recruitment strategies.

## 1. Introduction

Cutaneous melanoma (CM) is the type of skin cancer associated with the highest burden of morbidity, mortality, and years of potential life lost [1]. However, CM can be largely prevented through patient education, sun protection, screening, and public health campaigns, as shown by Australia’s primary prevention efforts [2,3,4]. These comprehensive initiatives included multimedia campaigns, as well as educational, organizational, and environmental strategies and policies, which have led to well-documented impacts on sun protective behaviors [2,5,6,7,8,9,10,11,12,13,14,15,16,17]. Their positive impact was further highlighted by a plateau in the incidence of CM in the mid 1990s and a CM incidence decline in individuals between the ages of 15 and 24 years [10]. One of the critical steps in the success of these campaigns was the incorporation of research and evaluation into the planning, implementation, and development stages of the programs [13].

In recent decades, incidence rates of CM have been rising globally and have become a public health concern worldwide, including in North America [18,19,20]. Previous studies led by our research group have demonstrated that the overall incidence rate per 100,000 individuals per year in Canada increased from 12.29 during 1992–2010 to 20.75 during 2011–2017 [21]. Differences between Canadian provinces were noted: Prince Edward Island (PEI) and Nova Scotia (NS) have the highest age-standardized incidence, with 30.94 and 27.66 cases per 100,000 person-years, respectively [21]. In contrast, other Canadian Atlantic provinces, such as Newfoundland and Labrador (NL) and New Brunswick (NB), were found to have age-standardized incidence rates below or comparable to the Canadian average (16.63 and 19.99 per 10,000 person-years), respectively [21].

Atlantic Canada is an important unit in North America and multiple regional differences in CM incidence rates exist within this region, as detailed in several papers [21,22,23] and summarized graphically in Supplementary Figure S1. While the Western Canada Melanoma Study Group has published several papers assessing the association between various risk factors and the development of CM in Western Canada [24,25,26], to our knowledge, no previous large-scale study has assessed CM risk factors in the Canadian Atlantic region. The overall goal of this study is to compare UV exposure, sun protective behaviors, level of worry and baseline CM knowledge in high- versus low-incidence areas for CM in Atlantic Canada, as well as between various demographic groups based on income, education, sexual orientation and gender, with the intention of informing future primary prevention efforts.

## 2. Methods

The study design is reported in accordance with the Checklist for Reporting Results of Internet E-Surveys (CHERRIES) [27].

### 2.1. Study Design

We conducted a survey-based cross-sectional study in Canadian Atlantic provinces. A sample size calculation with a 95% confidence level and a 5% margin of error showed that a sample of approximately 384 individuals per province in each of the Atlantic provinces (PEI, NS, NL, NB) was sufficient to be representative of the population. However, given that we were planning to stratify variables based on a variety of parameters (see below), we anticipated to recruit over 5000 participants to ensure an adequate number of participants per group (actual recruitment *n* = 7861 participants).

### 2.2. Ethics Statement

The study protocol was approved by the research ethics boards of McGill University (Study Number A04-B16-20B) and Dalhousie University (Study #5721), as well as the Atlantic PATH project review committee.

Informed consent was obtained from all participants electronically prior to completing the survey. Participants were informed about the length of time of the survey, details on data storage, principal investigators, and study purpose. Data were stored on a secure network, which was only accessible to members of the research team via two-factor authentication, in compliance with General Data Protection Regulations principles.

### 2.3. Development and Pre-Testing

Participants were invited to complete an electronic validated patient questionnaire, the Sun Exposure and Behavior Inventory (SEBI), which has been shown to be valid and reliable [28]. The survey was expanded to include more demographic variables, as well as more detailed questions on UV exposure, sun protection practices, CM knowledge and the level of worry for CM. We also worked in collaboration with a research team, MaelStrom Research, to ensure that the wording of the questions in the survey was clear, direct and objective. Prior to recruitment, the survey was heavily tested by multiple users to ensure that there were no technical errors in the survey or in the embedded conditional functions.

### 2.4. Recruitment Process and Survey Administration

Participants were recruited on a volunteer basis between July 2020 and August 2022 through our partnership with *Atlantic PATH,* which is a pre-existing patient cohort with participants from NS, NB, PEI and NL (https://www.atlanticpath.ca/index.php/about-us/about-our-participants/, accessed on 15 June 2022). *Atlantic PATH* is funded by the Canadian Partnership Against Cancer. Recruitment strategies for this cohort included, but were not limited to, advertisement, workspace and community events, community leaders promoting participation, outreach activities, media coverage and invitations from provincial insurance programs (in NS) [29]. The *Atlantic PATH* cohort was selected because it addresses the gap in large-scale data focusing on chronic diseases and cancer within Atlantic provinces, and because it is part of largest chronic disease research project partnership in Canada. Inclusion criteria were ages 30 to 74 years and residency in an Atlantic province (NS, NB, PEI and NL). A total of 31,173 participants were recruited between 2009 and 2015 in the *Atlantic PATH* harmonized dataset. Recruited individuals were mostly female (70%), and 63% were above the age of 50 years [29]. The cohort’s province of residency repartition versus the estimated representation (provided by Statistics Canada) of the population in a given province were as follows: 53% vs. 40% in NS; 28% vs. 32% in NB; 4% vs. 6% in in PEI; 15% vs. 22% in NL. In total, 25,669 personal invitations were sent by email to participants of the *Atlantic Path* cohort.

Other recruitment strategies for this study included newsletters, which were sent to all participants who had completed the survey and agreed to be recontacted, to invite them to ask friends and family members to complete the survey through our online portal (https://portal.rimuhc.ca/cim/redcap/surveys/?s=MHH8PJ7CC9, developed by our team in June 2020). Adaptive questioning was used throughout the survey to ensure data quality. To ensure survey completeness, items required an answer from the participant, and non-response options (e.g., “not applicable”, “would rather not say”) were available. Responders were able to change their answers prior to submission by clicking the “previous page” button.

### 2.5. Analysis

Participants that were younger than 16 years or had incomplete survey responses were excluded. In total, 8312 individuals from Atlantic Canada agreed to participate in the study. Of these, 56 (0.7%) were excluded because they did not meet the age requirement or entered invalid ages, 385 (4.6%) were excluded due to incomplete surveys, and 7861 (94.6%) were included in the final analysis. In this study, 19 out of the 42 questionnaire items were assessed, which included demographic questions, as well as questions on UV exposure and skin cancer history, sun protection and level of worry.

General baseline demographic variables and survey responses were summarized by province using frequency counts and/or percentages for categorical variables and means (standard deviations) for continuous variables. For the comparisons, participants were divided based on region (PEI/NS vs. NL), annual household income after tax (≥50,000 $ vs. <50,000 $), education (university degree or higher vs. no university degree), self-reported identification with the LGBTQ+ community and gender. The high-(PEI/NS) and low-incidence (NL) groups had a statistically higher and lower CM incidence than the national average, respectively. On the other hand, NB (CM incidence rate 19.99, 95% CI: 18.85–21.13) was not significantly different from the national average (20.75, 95%CI: 20.54–20.95) and therefore not included in the regional comparison. Categorical variables were compared between groups using the Z-test for equality of proportions. Crude odds ratios with their corresponding 95% confidence intervals for the exposures of interest were calculated. In addition, logistic regression analyses were performed, and gender- and age-adjusted odds ratios were reported. *p*-values < 0.05 were considered to be statistically significant.

## 3. Results

### 3.1. Participants and Descriptive Data

A total of 7861 participants were included in the study, with 1048 from NL, 4217 from NS, 2242 from NB and 354 from PEI. Men represented 28.0% of participants and the mean age was 61.3 years (standard deviation = 9.8). Most participants (95.7%) were of non-Hispanic white or European/Canadian descent, who are most at risk of developing a skin cancer in their lifetime [30]. The response rate for individuals recruited through *Atlantic PATH* was approximately 28% (6347 out of 22,669 participants with active email addresses); however, an overall response rate could not be calculated given the open nature of our recruitment strategies. Detailed participant characteristics by province are listed in Supplementary Table S1. Information on overall UV exposure and risk factors, level of worry and CM knowledge by province is provided in Supplementary Tables S2–S4.

### 3.2. High-Incidence vs. Low-Incidence Regions for Melanoma

**Figure 1:**
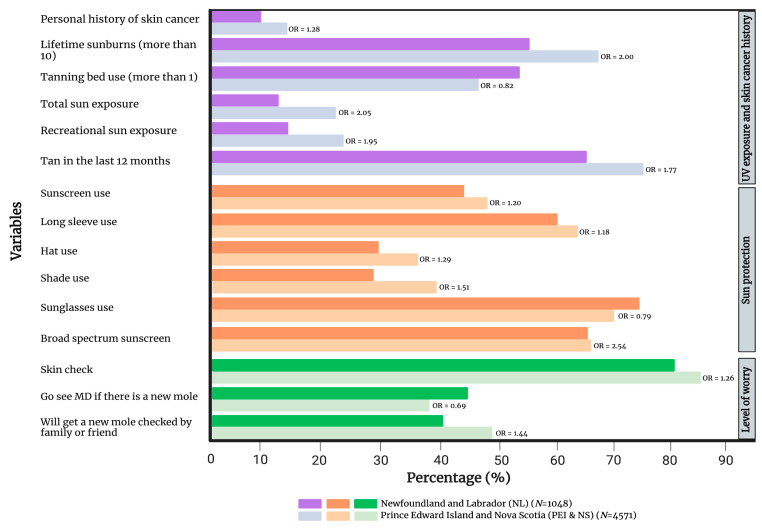
Comparison of UV exposure and skin cancer history, sun protection and level of worry between high-incidence (PEI/NS) and low-incidence (NL) provinces for cutaneous melanoma. Only variables that have reached statistical significance are included in the figure. Supplementary Table S5 provides a complete list of variables. OR = gender- and age-adjusted odds ratio.

As expected, PEI and NS had a significantly higher self-reported personal history of skin cancer (OR 1.28, 1.02–1.60). In addition, PEI/NS had more lifetime sunburns (OR 2.00, 1.72–2.31), total sun exposure (OR 2.05, 1.68–2.50), recreational sun exposure (OR 1.95, 1.61–2.35) and tanning in the last 12 months (OR 1.77, 1.53–2.05) in comparison to NL. However, PEI/NS had significantly fewer tanning bed users (OR 0.82, 0.71–0.95). Interestingly, compared to NL, PEI/NS displayed more sun protective behaviors overall. Individuals were more likely to often or always use sunscreen (OR 1.20, 1.04–1.37), wear long-sleeve shirts (OR 1.18, 1.02–1.36), wear hats (OR 1.29, 1.11–1.49) and seek shade (OR 1.51, 1.30–1.75). However, they were less likely to wear sunglasses (OR 0.79, 0.67–0.92). Sunscreen users in PEI/NS were more likely to use products with a broad spectrum (OR 2.54, 1.32–4.89). Further, individuals in PEI/NS were more likely to check their skin for moles on a regular basis (OR 1.26, 1.06–1.51). As for reactions to new moles, PEI/NS participants were less likely to go see a family doctor (OR 0.69, 0.60–0.79), but were more likely to ask a friend or family member to check the new mole (OR 1.44, 1.25–1.66). A summary of the statistically significant differences between PEI/NS and NL is presented in Figure 1 and a comprehensive comparison is provided in Supplementary Table S5.

### 3.3. High- vs. Low-Income Participants

**Figure 2:**
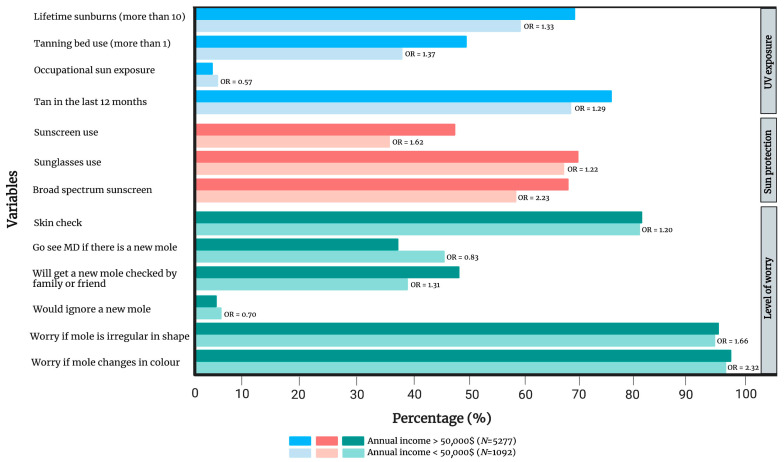
Comparison of UV exposure and skin cancer history, sun protection and level of worry between individuals with a lower income (< CAD 50,000 annually) and those with a higher income (>CAD 50,000 annually). Only variables that have reached statistical significance are included in the figure. Supplementary Table S6 provides a complete list of variables. OR = gender- and age-adjusted odds ratio.

Individuals with an annual income of CAD 50,000 or more (n = 5277) demonstrated overall more sun exposure, with a notable exception for occupational sun exposure (OR 0.57, 0.37–0.71) compared to individuals earning less than CAD 50,000 annually (n = 1092). Indeed, lifetime sunburns (OR 1.33, 1.15–1.54), tanning bed use (OR 1.37, 1.19–1.59) and having a tan in the last 12 months (OR 1.29, 1.11–1.49) were more frequent in the high-income group. In addition, they were more likely to use sun protection methods requiring an expense, such as wearing sunscreen (OR 1.62, 1.41–1.86), which was more frequently broad-spectrum (OR 2.23, 1.15–4.35), as well as sunglasses (OR 1.22, 1.06–1.41). Interestingly, individuals in the high-income group were less likely to go see a family doctor (OR 0.83, 0.73–0.96) and were more likely to ask a friend or a family member to check their new moles (OR 1.31, 1.14–1.50). They were also more likely to perform self-skin checks (OR 1.20, 1.01–1.44) and would be more worried if a mole changed in shape (OR 1.66, 1.02–2.72) or colour (OR 2.32, 1.30–4.17). Further information is presented in Figure 2 and Supplementary Table S6.

### 3.4. Highest Level of Education Completed

**Figure 3:**
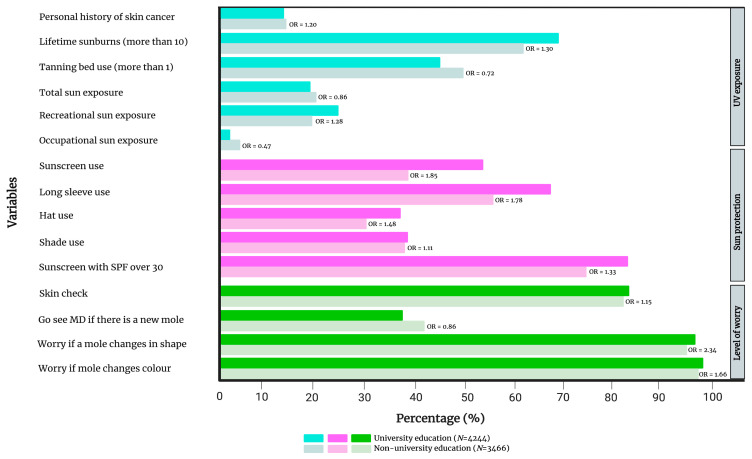
Comparison of UV exposure and skin cancer history, sun protection and level of worry between individuals with and without a university education. Only variables that have reached statistical significance are included in the figure. Supplementary Table S7 provides a complete list of variables. OR = gender- and age-adjusted odds ratio.

There were many parallels between individuals with a university degree (n = 4244) and those earning an annual income ≥ CAD 50,000. Indeed, those with a university training also reported more lifetime sunburns (OR 1.30, 1.17–1.44) in addition to higher rates of recreational sun exposure (OR 1.28, 1.15–1.43), while occupational sun exposure was less frequent (OR 0.47, 0.36–0.60) when compared to the group without a university degree (n = 3466). Individuals with a university degree exhibited more overall sun protective behaviours with more sunscreen use (OR 1.85, 1.68–2.03), long-sleeve use (OR 1.78, 1.61–1.96), hat use (OR 1.48–1.34–1.63), shade use (OR 1.11, 1.01–1.22) and SPF 30+ sunscreen usage (OR 1.33, 1.14–1.54). Interestingly, individuals with a university education were less likely to use a tanning bed (OR 0.72, 0.66–0.80) and had less total sun exposure (OR 0.86, 0.77–0.96). Individuals with a university degree were more worried compared to the group without a university degree if a mole was irregular in shape (OR 2.34, 1.58–3.44) or colour (OR 1.66, 1.03–2.65). Similarly to the high-income group, those with a university degree were more likely to perform regular skin checks (OR 1.15, 1.02–1.31), but were less likely to have a new mole inspected by a family physician (OR 0.86, 0.78–0.94). The statistically significant differences are summarized in Figure 3 and the complete list of comparisons is presented in Supplementary Table S7.

### 3.5. Sexual Orientation and Gender

**Figure 4:**
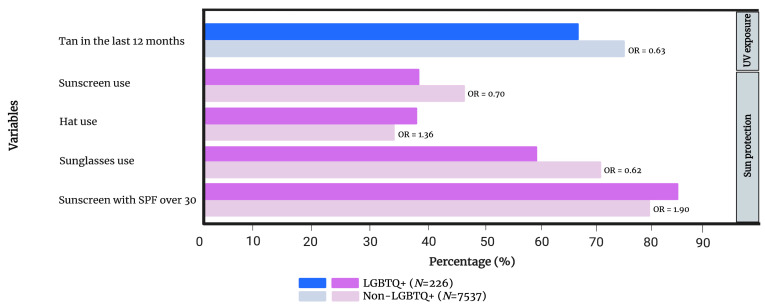
Comparison of UV exposure and skin cancer history, sun protection and level of worry between individuals who self-identify with the LGBTQ+ community and those who do not. Only variables that have reached statistical significance are included in the figure. Supplementary Table S8 provides a complete list of variables. OR = gender- and age-adjusted odds ratio.

No significant differences were found in lifetime sunburns, lifetime blistering sunburns, tanning bed use, total/recreational/occupational sun exposure, reaction to a new mole or level of worry between the self-identified LGBTQ+ (n = 266) vs. non-LGBTQ+ (n = 7537) populations of our cohort. Regarding sun exposure, individuals identifying with the LGBTQ+ community were less likely to report a tan in the last 12 months (OR 0.63, 0.47–0.84). The LGBTQ+ population wore more hats (OR 1.36, 1.03–1.81), but less sunscreen (OR 0.70, 0.53–0.93) and sunglasses (OR 0.62, 0.47–0.82). While members of the LGBTQ+ community were less likely to wear sunscreen, those that did were more likely to use an SPF 30+ (OR 1.90, 1.06–3.43). Additional information is presented in Figure 4 and Supplementary Table S8.

**Figure 5:**
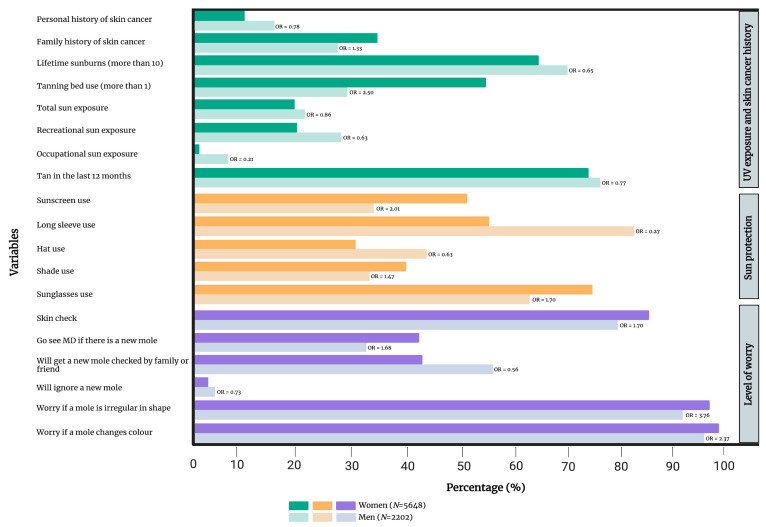
Comparison of UV exposure and skin cancer history, sun protection and level of worry between men and women. Only variables that have reached statistical significance are included in the figure. Supplementary Table S9 provides a complete list of variables. OR = gender- and age-adjusted odds ratio.

With respect to gender, women (n = 5648) were less likely to report ≥10 lifetime sunburns (OR 0.65, 0.58–0.73), high or very high total sun exposure (OR 0.86, 0.76–0.97), recreational sun exposure (OR 0.63, 0.56–0.71), occupational sun exposure (OR 0.21, 0.16–0.27) and having a tan in the last 12 months (OR 0.77, 0.69–0.87) compared to men (n = 2202). Women also reported significantly less long-sleeve use (OR 0.27, 0.24–0.31) and hat use (OR 0.63, 0.57–0.70). On the other hand, women reported significantly more tanning bed use (OR 2.50, 2.25–2.79), sunscreen use (OR 2.01, 1.81–2.23), shade seeking (OR 1.47, 1.32–1.64) and sunglass wearing (OR 1.70, 1.53–1.89). Additionally, women were significantly more likely to check their skin for new moles (OR 1.70, 1.49–1.93) and were more likely to be worried if a mole changed in shape (OR 3.76, 2.61–5.42) or colour (OR 2.37, 1.50–3.75). Significantly more women would consult a family doctor if they noticed a new mole (OR 1.68, 1.51–1.87). On the other hand, less women would have a family member or friend check their mole (OR 0.56, 0.51–0.62) or would ignore it (OR 0.73, 0.58–0.93) compared to men. More details are presented in Figure 5 and Supplementary Table S9.

## 4. Discussion

To our knowledge, this is the first large-scale, cross-sectional study investigating skin cancer risk factors, baseline melanoma knowledge, sun protection behaviors, as well as the level of worry in Atlantic Canada. Of note, international cross-sectional studies have included Canadian respondents; however, details on Canadian participants were not reported individually [31]. With regards to high-(PEI/NS) and low-incidence (NL) regions, individuals from PEI/NS are noted to have more overall sun exposure. PEI/NS have the greatest percentage of individuals with Fitzpatrick skin type I–II that have a predisposition to burn, related to the high number of individuals with a British Isles background in these provinces [22,32]. Together, these factors likely contribute to the higher burden of CM observed in these provinces. Furthermore, the findings of our study suggest that increased sun exposure, rather than low awareness or lower use of sun protection, is likely driving the higher rates of CM observed in PEI/NS compared to NL. We believe these results offer insight into the high degree of variability in sun safety awareness, knowledge and behaviors between geographic regions. As such, our findings may benefit international regions with similar demographics by identifying gaps in local photoprotective behaviors and public awareness, in hopes of guiding future primary prevention strategies. Examples of effective interventions from the Australian model that have focused on reducing UV exposure include bans on commercial solariums, policies and procedures in early childhood services and schools, sun protection within sports and recreation, and finally, shade development for local government buildings, facilities, community events and outdoor work/activities [33,34,35,36,37,38]. These strategies could be tailored and adapted to the population in Atlantic Canada, with the intention of achieving similar successes in the decades to come.

Previous studies have shown that a high socioeconomic status (SES) is associated with an increased risk of CM [25,39,40,41]. Based on our results, risk factors contributing to this increased risk include more lifetime sun burns, tanning bed use and being tanned. A higher SES is known to be associated with more vacations in sunny climates and recreational tanning, which supports these findings [40,42]. A Danish study also indicated that tanning bed use was linked to a higher income, which corroborates our findings [43]. Interestingly, a Northern European survey highlighted that despite sunscreen sales being more prevalent in high-income countries, they still have higher rates of CM [41]. These findings suggest that higher UV exposure may be driving the higher rates of CM observed in higher SES groups. On the other hand, findings from a German cohort study indicate that children from lower-income households (<60% of median) were significantly less likely to protect themselves with sunglasses. The authors found no further significant discrepancies in sun protective behaviors in relation to SES [44]. Our results show that individuals who earn <CAD 50,000 annually have significantly more occupational sun exposure. There exists an association between SES and working conditions, whereby outdoor workers are more frequently in disadvantaged socioeconomic classes and at a higher risk of sun exposure [45]. Regarding sun protection, our study shows that costly sun protective measures (e.g., sunscreen, sunglasses) are more frequently practiced in the high-income group. While the trends based on the level of education are mostly parallel to those based on income, an important difference is that the high-income group has more tanning bed users, whereas the opposite holds true in the university-educated group. Together, these findings suggest that establishing policies to protect outdoor workers, having sun protective measures (especially sun protective clothing) that are more financially accessible and increasing awareness on the dangers of tanning bed/UV exposure could help reduce CM risk.

An American study investigating skin cancer risk factors found that sexual-minority men were more likely to use tanning beds and had a higher lifetime skin cancer risk than heterosexual men, whereas women belonging to a sexual minority were less likely to use tanning beds than their heterosexual counterparts [46]. Interestingly, our study did not find any significant differences in self-reported history of skin cancer, lifetime sunburns, sun exposure or tanning bed use between individuals that identify with the LGBTQ+ community in Atlantic Canada and those that do not. More studies are needed to clarify risk factors that are specific to this population.

Results stratified by gender show that, overall, women have less sun exposure and practice more sun protection compared to men, with the exception that they wear less long-sleeve shirts and more frequently use tanning beds. This may explain our prior findings of Canadian women being more likely to develop melanoma on their extremities [22]. Also, tanning bed use early in life may explain the higher rates of melanoma in Canadian women < 40 [22,23,47]. Regarding awareness, a prior study found that men express more negative beliefs toward sunscreen use than women, whereas women are more likely to acknowledge its beneficial impact (especially the anti-UVA properties which help prevent photoaging) [48]. Consistent with this, women were more concerned about new moles or moles changing in shape and were more likely to seek consultation with a family physician. These behavior patterns may account for the overall lower melanoma incidence and mortality in women [21,22,23,47].

### 4.1. Strengths

The strengths of this study include its in-depth view of skin cancer prevention and sun safety behaviors in Atlantic Canada across a variety of variables, the large cohort of responders and the use of a modified validated patient questionnaire.

### 4.2. Limitations

This study also had limitations. Our results were subject to recall bias, as well as participation bias since participants were mostly recruited through the *Atlantic PATH* cohort, which is not representative of the general population in Atlantic Canada. For example, younger adults and men were underrepresented. While men were underrepresented in our study (28%), our proportion is similar to the *Atlantic PATH* cohort’s gender ratio (30% men) [29]. Similarly, the average age of our cohort mirrors that of the *Atlantic PATH cohort.* It is conceivable that *Atlantic PATH’s* recruitment strategies have created a cohort with a higher prevalence of personal and familial history of chronic conditions and cancer. Together, these factors could have biased our results and impacted their generalizability. For example, individuals with a personal or family history of skin cancer may expose themselves less to the sun and practice more sun-protective behaviours. Similarly, sun-protective practices could significantly vary among younger age groups. For example, large-scale cross-sectional analyses focusing on young children and adolescents have been published and show that this age group practices inadequate sun protection and has a low awareness of skin cancer [49]. While our survey targeted adult participants, similar themes have been found in younger individuals across studies, highlighting the need for more sun protection and education [50,51,52]. Moreover, most participants were non-Hispanic white or European/Canadian descent, which is reflective of the ethnic/racial composition of these provinces. Ethnicity and skin color, which could influence UV exposure and sun protection patterns, were not assessed due to the low number of non-white participants. While gender- and age-adjusted ORs were calculated, other confounding factors were not accounted for, and multivariate analyses were not performed. The results of survey studies do not establish causation, but rather correlation [10]. The survey study also had a low response rate. It is possible that the contact information of certain *Atlantic PATH* participants may be inaccurate, which could have led to an underestimation of the response rate. Lastly, some participants answered “I do not know” or “I would rather not say” to certain questions, which could have biased the results toward more conservative answers.

## 5. Conclusions

In conclusion, the findings of this study highlight important geographic and demographic differences that could help guide public health interventions related to sun protection/melanoma awareness tailored to the target population in a given region.

## Data Availability

All available data is presented in the text of the manuscript and in Supplementary Materials.

## Supplementary Materials

The following supporting information can be downloaded at: https://www.mdpi.com/article/10.3390/cancers15153753/s1, Figure S1: Melanoma incidence trends by forward sortation area (FSA—first three entries of a postal code) in Atlantic Canada. Geographic maps illustrate incidence rates for cutaneous melanoma (cases per 100,000 individuals per year) relative to the national average based on the Canadian Cancer Registry/Quebec Cancer Registry databases [21]. 1A: Newfoundland and Labrador 1B: High-incidence FSAs in the Maritime provinces with several high-incidence areas shown in Prince Edward Island, Nova Scotia and New Brunswick; Table S1: Population characteristics; Table S2: UV exposure, melanoma risk factors and sun protection; Table S3: Level of worry; Table S4: Melanoma knowledge—quotes; Table S5: Difference between high- and low-incidence regions; Table S6: Difference in proportions between populations with different income ranges; Table S7: Difference in proportions between populations with different education levels; Table S8: Difference between LGBTQ+ and non-LGBTQ+; Table S9: Difference between men and women.
