# Supplementary material for: Assessing Skin Cancer Risk Factors, Sun Safety Behaviors and Melanoma Concern in Atlantic Canada: A Comprehensive Survey Study
Source: Cancers (Basel). 2023 Jul 25;15(15):3753. doi: 10.3390/cancers15153753 (PMC10417242; doi:10.3390/cancers15153753)
Supplement: Supplementary file 1 [file cancers-15-03753-s001.zip › Supplementary Figure.pdf]

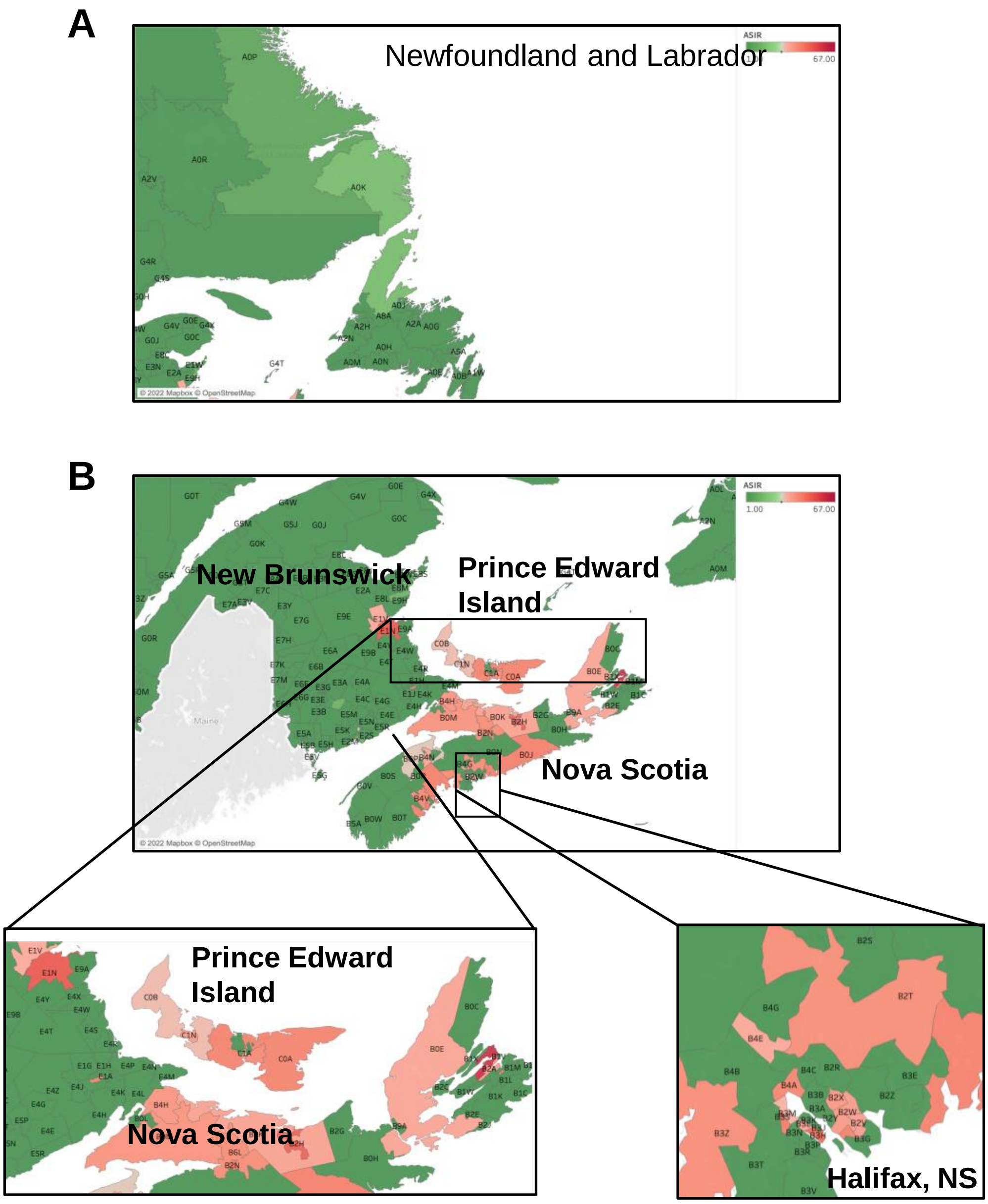

Figure S1

Melanoma incidence trends by forward sortation area (FSA—first three entries of a postal code) in Atlantic Canada. Geographic maps illustrate incidence rates for cutaneous melanoma (cases per 100,000 individuals per year) relative to the national average based on the Canadian Cancer Reg-istry/Quebec Cancer Registry databases [21]. 1A: Newfoundland and Labrador 1B: High-incidence FSAs in the Maritime provinces with several high-incidence areas shown in Prince Edward Island, Nova Scotia and New Brunswick.
