# Supplementary material for: Assessing Skin Cancer Risk Factors, Sun Safety Behaviors and Melanoma Concern in Atlantic Canada: A Comprehensive Survey Study
Source: Cancers (Basel). 2023 Jul 25;15(15):3753. doi: 10.3390/cancers15153753 (PMC10417242; doi:10.3390/cancers15153753)
Supplement: Supplementary file 1 [file cancers-15-03753-s001.zip › Supplementary Tables.pdf]

**TABLE S1: POPULATION CHARACTERISTICS**

**Participants could select more than one answer

| Variable (n, %) / Province | Newfoundland (NL) N= 1048 | Nova Scotia (NS) N= 4217 | New Brunswick (NB) N= 2242 | Prince Edward Island (PEI) N= 354 | Atlantic Provinces N=7861 |
|---|---|---|---|---|---|
| ***Age (mean, standard deviation)*** | 60.0 (9.8) | 62.4 (9.6) | 59.9 (10.1) | 62.2 (9.5) | 61.3 (9.8) |
| ***Gender (% men)*** | 319 (30.4%) | 1190 (28.2%) | 613 (27.8%) | 80 (22.6%) | 2202 (28.0%) |
| ***Ethnicity**, n (%)*** | | | | | |
| - Non-Hispanic White or Euro Canadian | 969 (92.5%) | 4044 (95.9%) | 2167 (96.7%) | 340 (96.1%) | 7520 (95.7%) |
| - Afro-Caribbean or African Canadian | 0 (-) | 19 (0.5%) | 5 (0.2%) | 2 (0.6%) | 26 (0.3%) |
| - Latino or Hispanic Canadian | 2 (0.2%) | 11 (0.3%) | 2 (0.1%) | 0 (-) | 15 (0.2%) |
| - East Asian or Asian Canadian | 0 (-) | 12 (0.3%) | 5 (0.2%) | 0 (-) | 17 (0.2%) |
| - South Asian or Indian Canadian | 0 (-) | 12 (0.3%) | 6 (0.3%) | 0 (-) | 18 (0.2%) |
| - Middle Eastern or Arab Canadian | 5 (0.5%) | 19 (0.5%) | 5 (0.2%) | 2 (0.6%) | 31 (0.4%) |
| - Indigenous | 57 (5.4%) | 59 (1.4%) | 26 (1.2%) | 2 (0.6%) | 144 (1.8%) |
| - Other | 35 (3.3%) | 118 (2.8%) | 58 (2.6%) | 14 (4.0%) | 225 (2.9%) |
| ***Income, n (%)*** | | | | | |
| - < 20 000$ | 14 (1.3%) | 63 (1.5%) | 29 (1.3%) | 6 (1.7%) | 112 (1.4%) |
| - 20 000-49 999$ | 115 (11.0%) | 554 (13.1%) | 252 (11.2%) | 59 (16.7%) | 980 (12.5%) |
| - 50 000-69 999$ | 174 (16.6%) | 707 (16.8%) | 361 (16.1%) | 72 (20.3%) | 1314 (16.7%) |
| - 70 000-89 999$ | 152 (14.5%) | 692 (16.4%) | 357 (15.9%) | 38 (10.7%) | 1239 (15.8%) |
| - ≥90,000$ | 396 (37.8%) | 1435 (34.0%) | 780 (34.8%) | 113 (31.9%) | 2724 (34.7%) |
| ***Highest level of education completed, n (%)*** | | | | | |
| - No high school | 6 (0.6%) | 46 (1.1%) | 27 (1.2%) | 2 (0.6%) | 81 (1.0%) |
| - High school | 119 (11.4%) | 615 (14.6%) | 300 (13.4%) | 56 (15.8%) | 1090 (13.9%) |
| - Cegep or college degree | 386 (36.9%) | 1141 (27.1%) | 650 (29.0%) | 118 (33.3%) | 2295 (29.2%) |
| - University Bachelor’s degree | 292 (27.9%) | 1279 (30.4%) | 739 (33.0%) | 112 (31.6%) | 2422 (30.8%) |
| - Graduate or doctoral studies | 219 (20.9%) | 1052 (25.0%) | 490 (21.9%) | 61 (17.2%) | 1822 (23.2%) |
| ***Eye colour, n (%)*** | | | | | |
| - Brown | 259 (24.7%) | 960 (22.8%) | 525 (23.4%) | 69 (19.5%) | 1813 (23.1%) |
| - Blue, green, or grey | 619 (59.1%) | 2343 (55.6%) | 1258 (56.1%) | 197 (55.7%) | 4417 (56.2%) |
| - Hazel (brownish green) | 170 (16.2%) | 914 (21.7%) | 459 (20.5%) | 88 (24.9%) | 1631 (20.8%) |
| ***Fitzpatrick, n (%)*** | | | | | |
| - Type I | 111 (10.6%) | 348 (8.3%) | 179 (8.0%) | 31 (8.8%) | 669 (8.5%) |
| - Type II | 294 (28.1%) | 1318 (31.3%) | 685 (30.6%) | 107 (30.2%) | 2404 (30.6%) |
| - Type III | 457 (43.6%) | 1802 (42.7%) | 991 (44.2%) | 150 (42.4%) | 3400 (43.3%) |
| - Type IV | 153 (14.6%) | 632 (15.0%) | 331 (14.8%) | 55 (15.5%) | 1171 (14.9%) |
| - Type V | 21 (2.0%) | 90 (2.1%) | 48 (2.1%) | 6 (1.7%) | 165 (2.1%) |
| - Type VI | 2 (0.2%) | 3 (0.1%) | 2 (0.1%) | 1 (0.3%) | 8 (0.1%) |

## TABLE S2: UV EXPOSURE, MELANOMA RISK FACTORS AND SUN PROTECTION

| Variable (n, %) / Province | Newfoundland (NL) N= 1048 | Nova Scotia (NS) N= 4217 | New Brunswick (NB) N= 2242 | Prince Edward Island (PEI) N= 354 | Atlantic Provinces N=7861 |
|---|---|---|---|---|---|
| **Personal history of skin cancer, *n (%)*** | 107 (10.2%) | 605 (14.4%) | 288 (12.9%) | 36 (10.2%) | 1036 (13.2%) |
| - Melanoma | 31 (29.0%) | 175 (28.9%) | 84 (29.2%) | 12 (33.3%) | 302 (29.2%) |
| - Squamous Cell Carcinoma (SCC) | 12 (11.2%) | 79 (13.1%) | 32 (11.1%) | 7 (19.4%) | 130 (12.6%) |
| - Basal Cell Carcinoma (BCC) | 57 (53.3%) | 289 (47.8%) | 130 (45.1%) | 17 (47.2%) | 493 (47.6%) |
| **Family history of skin cancer, *n (%)*** | 318 (30.3%) | 1427 (33.8%) | 699 (31.2%) | 104 (29.4%) | 2548 (32.4%) |
| **Lifetime sunburns (more than 10), *n (%)*** | 572 (54.6%) | 2843 (67.2%) | 1509 (67.3%) | 239 (67.5%) | 5154 (65.6%) |
| **Lifetime blistering sunburns (1 or more), *n (%)*** | 751 (71.7%) | 3007 (71.3%) | 1561 (69.6%) | 253 (71.5%) | 5572 (70.9%) |
| **Tanning bed use (more than 1), *n (%)*** | 555 (53.0%) | 1940 (46.0%) | 1046 (46.7%) | 176 (49.7%) | 3717 (47.3%) |
| **Sun exposure (high-very high), *n (%)*** | | | | | |
| - Total sun exposure | 130 (12.4%) | 935 (22.2%) | 468 (20.9%) | 79 (22.3%) | 1612 (20.5%) |
| - Recreational sun exposure | 148 (14.1%) | 1005 (23.8%) | 550 (24.5%) | 74 (20.9%) | 1777 (22.6%) |
| - Occupational sun exposure | 29 (2.8%) | 148 (3.5%) | 82 (3.7%) | 19 (5.4%) | 278 (3.5%) |
| **Tan in the last 12 months, *n (%)*** | 679 (64.8%) | 3157 (74.9%) | 1705 (76.1%) | 278 (78.5%) | 5819 (74.0%) |
| **Sun protection (often-always), *n (%)*** | | | | | |
| - Sunscreen use | 462 (44.1%) | 2013 (47.7%) | 998 (44.5%) | 177 (50.0%) | 3650 (46.4%) |
| - Long sleeve use | 629 (60.0%) | 2705 (64.2%) | 1355 (60.4%) | 214 (60.5%) | 4903 (62.4%) |
| - Hat use | 309 (29.5%) | 1519 (36.0%) | 720 (32.1%) | 146 (41.2%) | 2694 (34.3%) |
| - Shade use | 301 (28.7%) | 1670 (39.6%) | 875 (39.0%) | 135 (38.1%) | 2981 (37.9%) |
| - Sunglasses use | 779 (74.3%) | 2955 (70.1%) | 1606 (71.6%) | 242 (68.4%) | 5582 (71.0%) |
| **Sunscreen type, *n (%)*** | | | | | |
| - Broad-spectrum | 684 (65.3%) | 2811 (66.7%) | 1492 (66.6%) | 242 (68.4%) | 5229 (66.5%) |
| - SPF >30 | 818 (78.1%) | 3364 (79.8%) | 1791 (79.9%) | 278 (78.5%) | 6251 (79.5%) |

## TABLE S3: LEVEL OF WORRY

| Province / Variable (n, %) | Newfoundland (NL) N= 1048 | Nova Scotia (NS) N= 4217 | New Brunswick (NB) N= 2242 | Prince Edward Island (PEI) N= 354 | Atlantic Provinces N=7861 |
|---|---|---|---|---|---|
| **Skin Check, *n (%)*** | 854 (81.5%) | 3613 (85.7%) | 1807 (80.6%) | 289 (81.6%) | 6563 (83.5%) |
| **Reaction, *n (%)*** | | | | | |
| - Family doctor visit | 467 (44.6%) | 1620 (38.4%) | 865 (38.6%) | 146 (41.2%) | 3098 (39.4%) |
| - Check by friend/family member | 421 (40.2%) | 2026 (48.0%) | 1037 (46.3%) | 173 (48.9%) | 3657 (46.5%) |
| - Ignore | 47 (4.5%) | 183 (4.3%) | 96 (4.3%) | 9 (2.5%) | 335 (4.3%) |
| **Worry if mole, *n (%)*** | | | | | |
| - is irregular in shape | 997 (95.1%) | 4057 (96.2%) | 2167 (96.7%) | 348 (98.3%) | 7569 (96.3%) |
| - changes colour | 1022 (97.5%) | 4104 (97.3%) | 2197 (98.0%) | 351 (99.2%) | 7674 (97.6%) |
| - grew in size | 1025 (97.8%) | 4112 (97.5%) | 2190 (97.7%) | 348 (98.3%) | 7675 (97.6%) |

## TABLE S4: MELANOMA KNOWLEDGE - QUOTES

| Quotes (n, %) / Province | Newfoundland (NL) Low Incidence N= 1048 | Nova Scotia (NS) N= 4217 | New Brunswick (NB) N= 2242 | Prince Edward Island (PEI) N= 354 | Atlantic Provinces N=7861 | High Incidence (PEI/NS) N= 4571 |
|---|---|---|---|---|---|---|
| **Having a base tan is protective against the sun's UV/radiation/skin damage, *n (%)*** | | | | | | |
| - Agree | 136 (13.0%) | 627 (14.9%) | 305 (13.6%) | 58 (16.4%) | 1126 (14.3%) | 685 (15.0%) |
| - Strongly Agree | 16 (1.5%) | 43 (1.0%) | 24 (1.1%) | 5 (1.4%) | 88 (1.1%) | 48 (1.1%) |
| **It is rare to get melanoma before the age of 35, *n (%)*** | | | | | | |
| - Agree | 106 (10.1%) | 443 (10.5%) | 225 (10.0%) | 36 (10.2%) | 810 (10.3%) | 479 (10.5%) |
| - Strongly Agree | 9 (0.9%) | 40 (1.0%) | 17 (0.8%) | 1 (0.3%) | 67 (0.9%) | 41 (0.9%) |
| **I check my skin on a regular basis for abnormal moles, *n (%)*** | | | | | | |
| - Agree | 556 (53.1%) | 2321 (55.0%) | 1195 (53.3%) | 177 (50.0%) | 4294 (54.1%) | 2498 (54.7%) |
| - Strongly Agree | 169 (16.1%) | 706 (16.7%) | 339 (15.1%) | 63 (17.8%) | 1277 (16.2%) | 769 (16.8%) |
| **Sunscreens pollute the oceans, *n (%)*** | | | | | | |
| - Agree | 118 (11.3%) | 918 (21.8%) | 387 (17.3%) | 73 (20.6%) | 1496 (19.0%) | 991 (21.7%) |
| - Strongly Agree | 19 (1.8%) | 195 (4.6%) | 96 (4.3%) | 11 (3.1%) | 321 (4.1%) | 206 (4.5%) |
| **Sunscreens contain toxic ingredients, *n (%)*** | | | | | | |
| - Agree | 107 (10.2%) | 734 (17.4%) | 386 (17.2%) | 68 (19.2%) | 1295 (16.5%) | 802 (17.6%) |
| - Strongly Agree | 14 (1.3%) | 134 (3.2%) | 81 (3.6%) | 14 (4.0%) | 243 (3.1%) | 148 (3.2%) |
| **I look better/healthier with a tan, *n (%)*** | | | | | | |
| - Agree | 466 (44.5%) | 1947 (46.2%) | 1035 (46.2%) | 175 (49.4%) | 3623 (46.1%) | 2122 (46.4%) |
| - Strongly Agree | 77 (7.4%) | 230 (5.5%) | 120 (5.4%) | 9 (2.5%) | 436 (5.6%) | 239 (5.2%) |
| **Tanning booths are a safer, more controlled way to get a tan than from the sun, *n (%)*** | | | | | | |
| - Agree | 11 (1.1%) | 63 (1.5%) | 31 (1.4%) | 4 (1.1%) | 109 (1.4%) | 67 (1.5%) |
| - Strongly Agree | 16 (1.5%) | 47 (1.1%) | 23 (1.0%) | 5 (1.4%) | 91 (1.2%) | 52 (1.1%) |

**TABLE S5: DIFFERENCE BETWEEN HIGH AND LOW INCIDENCE REGIONS.**

| Variable (n, %) | High Incidence (PEI/NS) N=4571 | Low Incidence (NL) N=1048 | Difference** (95% CI) | Odds Ratio (95% CI) | Adjusted Odds Ratio+ (95% CI) | P-value of Adjusted Odds Ratio |
|---|---|---|---|---|---|---|
| **Personal history of skin cancer** | 641 (14.0%) | 107 (10.2%) | 3.8% (1.7%-5.9%) | 1.4 (1.2-1.8) | 1.28 (1.02-1.60) * | 0.03 |
| - Melanoma | 187 (29.2%) | 31 (29.0%) | 0.2% (-9.1%-9.5%) | 1.0 (0.6-1.6) | | |
| - Squamous Cell Carcinoma | 86 (13.4%) | 12 (11.2%) | 2.2% (-4.3%-8.7%) | 1.2 (0.6-2.6) | | |
| - Basal Cell Carcinoma | 306 (47.7%) | 57 (53.3%) | -5.5% (-15.7%-4.7%) | 0.8 (0.5-1.2) | | |
| **Family history of skin cancer** | 1531 (33.5%) | 318 (30.3%) | 3.2% (0.1%-6.3%) | 1.2 (1.0-1.3) | 1.16 (1.00-1.34) | 0.05 |
| **Lifetime sunburns (more than 10)** | 3073 (67.2%) | 572 (54.6%) | 12.6% (9.3%-16.0%) | 1.7 (1.5-2.0) | 2.00 (1.72 – 2.31) * | <0.001 |
| **Lifetime blistering sunburns (more than 1)** | 3260 (71.3%) | 751 (71.7%) | -0.3% (-3.3%-2.7%) | 0.9 (0.8-1.1) | 0.95 (0.81-1.12) | 0.58 |
| **Tanning bed use (more than 1)** | 2116 (46.3%) | 555 (53.0%) | -6.7% (-10.0%- -3.3%) | 0.8 (0.7-0.9) | 0.82 (0.71-0.95) * | 0.008 |
| **Sun exposure (high-very high)** | | | | | | |
| - Total sun exposure | 1014 (22.2%) | 130 (12.4%) | 9.8% (7.4%-12.1%) | 2.0 (1.7-2.5) | 2.05 (1.68-2.50) * | <0.001 |
| - Recreational sun exposure | 1079 (23.6%) | 148 (14.1%) | 9.5% (7.0%-11.9%) | 1.9 (1.6-2.3) | 1.95 (1.61-2.35) * | <0.001 |
| - Occupational sun exposure | 167 (3.7%) | 29 (2.8%) | 0.9% (-0.2%-2.0%) | 1.3 (0.9-2.1) | 1.47 (0.98-2.21) | 0.06 |
| **Tan in the last 12 months** | 3435 (75.2%) | 679 (64.8%) | 10.4% (7.2%-13.5%) | 1.6 (1.4-1.9) | 1.77 (1.53-2.05) * | <0.001 |
| **Sun protection (often-always)** | | | | | | |
| - Sunscreen use | 2190 (47.9%) | 462 (44.1%) | 3.8% (0.5%-7.2%) | 1.2 (1.0-1.3) | 1.20 (1.04-1.37) * | 0.01 |
| - Long sleeve use | 2919 (63.9%) | 629 (60.0%) | 3.8% (0.6%- 7.1%) | 1.2 (1.0-1.4) | 1.18 (1.02-1.36) * | 0.02 |
| - Hat use | 1665 (36.4%) | 309 (29.5%) | 6.9% (3.8%-10.0%) | 1.4 (1.2-1.6) | 1.29 (1.11-1.49) * | 0.001 |
| - Shade use | 1805 (39.5%) | 301 (28.7%) | 10.8% (7.7%-13.9%) | 1.6 (1.4-1.9) | 1.51 (1.30-1.75) * | <0.001 |
| - Sunglasses use | 3197 (69.9%) | 779 (74.3%) | 21.2% (18.8%-23.6%) | 0.8 (0.7-0.9) | 0.79 (0.67-0.92) * | 0.002 |
| **Sunscreen type** | | | | | | |
| - Broad-spectrum | 3053 (66.8%) | 684 (65.3%) | 1.5% (-1.7%-4.7%) | 1.1 (0.9-1.2) | 2.54 (1.32-4.89) * | 0.005 |
| - SPF >30 | 3642 (79.7%) | 818 (78.1%) | 1.6% (-1.1%-4.4%) | 1.1 (0.9-1.3) | 1.02 (0.81-1.29) | 0.85 |
| **Skin Check** | 3902 (85.4%) | 854 (81.5%) | 3.9% (1.3%-6.4%) | 1.3 (1.1-1.6) | 1.26 (1.06-1.51) * | 0.01 |
| **Reaction** | | | | | | |
| - Family doctor visit | 1766 (38.6%) | 467 (44.6%) | -5.9% (-9.3%- -2.6%) | 0.8 (0.7-0.9) | 0.69 (0.60-0.79) * | <0.001 |
| - Check by friend/family member | 2199 (48.1%) | 421 (40.2%) | 7.9% (4.6%-11.2%) | 1.4 (1.2-1.6) | 1.44 (1.25-1.66) * | <0.001 |
| - Ignore | 192 (4.2%) | 47 (4.5%) | -0.3% (-1.7%-1.1%) | 0.9 (0.7-1.3) | 0.93 (0.67-1.30) | 0.69 |
| **Worry if mole** | | | | | | |
| - is irregular in shape | 4405 (96.4%) | 997 (95.1%) | 1.2% (-0.2%-2.6%) | 1.4 (1.0-1.9) | 1.09 (0.65-1.82) | 0.75 |
| - changes colour | 4455 (97.5%) | 1022 (97.5%) | -0.06% (-1.1%-1.0%) | 1.0 (0.6-1.5) | 0.79 (0.39-1.63) | 0.53 |
| - grew in size | 4460 (97.6%) | 1025 (97.8%) | -0.2% (-1.2%-0.8%) | 0.9 (0.5-1.4) | 0.73 (0.37-1.45) | 0.37 |

*Statistically significant

**Percent difference between high incidence and low incidence provinces

+Gender and age-adjusted odds ratios

**TABLE S6: DIFFERENCE IN PROPORTIONS BETWEEN POPULATIONS WITH DIFFERENT INCOME RANGES.**

| Variable (n,%) | Income > 50K N=5277 | Income < 50K N=1092 | Difference (95% CI, p-value) | Odds Ratio (95% CI) | Adjusted Odds Ratio (95% CI) | P-value of Adjusted Odds Ratio |
|---|---|---|---|---|---|---|
| **Personal history of skin cancer** | 670 (12.7%) | 149 (13.6%) | -0.9% (-3.2%-1.3%) | 0.9 (0.8-1.1) | 1.21 (0.99-1.48) | 0.06 |
| - Melanoma | 195 (29.1%) | 42 (28.2%) | 0.9% (-7.1%-8.9%) | 1.0 (0.7-1.6) | | |
| - Squamous Cell Carcinoma | 84 (12.5%) | 16 (10.7%) | 1.8% (-3.8%-7.4%) | 1.2 (0.7-2.3) | | |
| - Basal Cell Carcinoma | 323 (48.2%) | 72 (48.3%) | -0.1% (-9.0%-8.8%) | 1.0 (0.7-1.4) | | |
| **Family history of skin cancer** | 1706 (32.3%) | 360 (33.0%) | -0.6% (-3.7%-2.4%) | 1.0 (0.8-1.1) | 1.04 (0.90-1.21) | 0.57 |
| **Lifetime sunburns (more than 10)** | 3656 (69.3%) | 641 (58.7%) | 10.6% (7.4%-13.8%) | 1.6 (1.4-1.8) | 1.33 (1.15-1.54) * | <0.001 |
| **Lifetime blistering sunburns (more than 1)** | 3813 (72.3%) | 763 (69.9%) | 2.4% (-0.6%- 5.4%) | 1.1 (1.0-1.3) | 1.06 (0.91-1.24) | 0.47 |
| **Tanning bed use (more than 1)** | 2603 (49.3%) | 413 (37.8%) | 11.5% (8.3%-14.7%) | 1.6 (1.4-1.8) | 1.37 (1.19-1.59) * | <0.001 |
| **Sun exposure (high-very high)** | | | | | | |
| - Total sun exposure | 1063 (20.1%) | 220 (20.2%) | 0.0% (-2.6%-2.6%) | 1.0 (0.8-1.2) | 0.97 (0.82-1.15) | 0.72 |
| - Recreational sun exposure | 1227 (23.3%) | 215 (19.7%) | 3.6% (0.9%-6.2%) | 1.2 (1.0-1.5) | 1.14 (0.96-1.35) | 0.13 |
| - Occupational sun exposure | 182 (3.5%) | 57 (5.2%) | -1.8% (-3.2%- -0.4%) | 0.6 (0.5-0.9) | 0.57 (0.37-0.71) * | <0.001 |
| **Tan in the last 12 months** | 4018 (76.1%) | 750 (68.7%) | 7.5% (4.5%-10.4%) | 1.5 (1.3-1.7) | 1.29 (1.11-1.49) * | <0.001 |
| **Sun protection (often-always)** | | | | | | |
| - Sunscreen use | 2514 (47.6%) | 395 (36.2%) | 11.5% (8.3%-14.6%) | 1.6 (1.4-1.8) | 1.62 (1.41-1.86) * | <0.001 |
| - Long sleeve use | 3336 (63.2%) | 658 (60.3%) | 3.0% (-0.2%- 6.1%) | 1.1 (1.0-1.3) | 1.14 (0.99-1.32) | 0.07 |
| - Hat use | 1760 (33.4%) | 372 (34.1%) | -0.7% (-3.8%-2.4%) | 1.0 (0.8-1.1) | 1.14 (0.98-1.31) | 0.08 |
| - Shade use | 1898 (36.0%) | 465 (42.6%) | -6.6% (-9.8%- -3.4%) | 0.8 (0.7-0.9) | 0.89 (0.78-1.02) | 0.10 |
| - Sunglasses use | 3710 (70.3%) | 734 (67.2%) | 3.1% (0.0%-6.1%) | 1.2 (1.0-1.3) | 1.22 (1.06-1.41) * | 0.007 |
| **Sunscreen type** | | | | | | |
| - Broad-spectrum | 3616 (68.5%) | 637 (58.3%) | 10.2% (7.0%-13.4%) | 1.6 (1.4-1.8) | 2.23 (1.15-4.35) * | <0.001 |
| - SPF >30 | 4266 (80.8%) | 786 (72.0%) | 8.9% (6.0%-11.7%) | 1.6 (1.4-1.9) | 0.93 (0.74-1.17) | 0.52 |
| **Skin Check** | 4372 (82.9%) | 901 (82.5%) | 0.3% (-2.1%- 2.8%) | 1.0 (0.9-1.2) | 1.20 (1.01-1.44) * | 0.04 |
| **Reaction** | | | | | | |
| - Family doctor visit | 1974 (37.4%) | 493 (45.2%) | -7.7% (-11.0%- -4.5%) | 0.7 (0.6-0.8) | 0.83 (0.73-0.96) * | 0.009 |
| - Check by friend/family member | 2559 (48.5%) | 426 (39.0%) | 9.5% (6.3%-12.7%) | 1.5 (1.3-1.7) | 1.31 (1.14-1.50) * | <0.001 |
| - Ignore | 229 (4.3%) | 58 (5.3%) | -1.0% (-2.4%- 0.5%) | 0.8 (0.6-1.1) | 0.70 (0.52-0.95) * | 0.002 |
| **Worry if mole** | | | | | | |
| - is irregular in shape | 5082 (96.3%) | 1043 (95.5%) | 0.8% (-0.5%- 2.1%) | 1.2 (0.9-1.7) | 1.66 (1.02-2.72) * | 0.04 |
| - changes colour | 5158 (97.7%) | 1056 (96.7%) | 1.0% (0.0%- 2.2%) | 1.5 (1.0-2.2) | 2.32 (1.30-4.17) * | 0.005 |
| - grew in size | 5150 (97.6%) | 1065 (97.5%) | 0.1% (-0.9%- 1.1%) | 1.0 (0.6-1.6) | 1.04 (0.55-1.95) | 0.91 |

*Statistically significant
**Percent difference between individuals with an annual income >50,000$ and those with an annual income <50,000$
+Gender and age-adjusted odds ratios

**TABLE S7: DIFFERENCE IN PROPORTION BETWEEN POPULATIONS WITH DIFFERENT EDUCATION LEVELS.**

| Variable (n,%) | University Education N=4244 | Non-University Education N= 3466 | Difference (95% CI, p-value) | Odds Ratio (95% CI) | Adjusted Odds Ratio (95% CI) | P-value of Adjusted Odds Ratio |
|---|---|---|---|---|---|---|
| **Personal history of skin cancer** | 578 (13.6%) | 506 (14.6%) | -1.0% (-2.5%-0.6%) | 0.9 (0.8-1.1) | 1.20 (1.04-1.37) * | 0.01 |
| - Melanoma | 160 (27.7%) | 162 (32.0%) | -4.3% (-10.0%-1.1%) | 0.8 (0.6-1.1) | | |
| - Squamous Cell Carcinoma | 69 (11.9%) | 63 (12.5%) | -0.5% (-4.4%-3.4%) | 1.0 (0.7-1.4) | | |
| - Basal Cell Carcinoma | 290 (50.2%) | 230 (45.5%) | 4.7% (-1.2%- 10.7%) | 1.2 (0.9-1.5) | | |
| **Family history of skin cancer** | 1382 (32.6%) | 1070 (30.8%) | 1.7% (-0.4%- 3.8%) | 1.1 (1.0-1.2) | 1.04 (0.94-1.14) | 0.49 |
| **Lifetime sunburns (more than 10)** | 2922 (68.9%) | 2143 (61.8%) | 7.0% (4.9%-9.2%) | 1.4 (1.2-1.5) | 1.30 (1.17-1.44) * | <0.001 |
| **Lifetime blistering sunburns (more than 1)** | 3018 (71.1%) | 2448 (70.6%) | 0.5% (-1.6%-2.5%) | 1.0 (0.9-1.1) | 1.00 (0.90-1.11) | 0.98 |
| **Tanning bed use (more than 1)** | 1911 (45.0%) | 1729 (49.9%) | -4.9% (-7.1%- -2.6%) | 0.8 (0.8-0.9) | 0.72 (0.66-0.80) * | <0.001 |
| **Sun exposure (high-very high)** | | | | | | |
| - Total sun exposure | 828 (19.5%) | 757 (21.8%) | -2.3% (-4.2%- -0.5%) | 0.9 (0.8-1.0) | 0.86 (0.77-0.96) * | 0.007 |
| - Recreational sun exposure | 1051 (24.8%) | 694 (20.0%) | 4.7% (2.9%- 6.6%) | 1.3 (1.2-1.5) | 1.28 (1.15-1.43) * | <0.001 |
| - Occupational sun exposure | 107 (2.5%) | 168 (4.9%) | -2.3% (-3.2%- -1.5%) | 0.5 (0.4-0.7) | 0.47 (0.36-0.60) * | <0.001 |
| **Tan in the last 12 months** | 3160 (74.5%) | 2569 (74.1%) | 0.3% (-1.6%- 2.3%) | 1.0 (0.9-1.1) | 0.97 (0.88-1.08) | 0.62 |
| **Sun protection (often-always)** | | | | | | |
| - Sunscreen use | 2256 (53.2%) | 1329 (38.3%) | 14.8% (12.6%-17.0%) | 1.8 (1.7-2.0) | 1.85 (1.68-2.03) * | <0.001 |
| - Long sleeve use | 2870 (67.2%) | 1930 (55.7%) | 11.9% (9.8%-14.1%) | 1.7 (1.5-1.8) | 1.78 (1.61-1.96) * | <0.001 |
| - Hat use | 1583 (37.3%) | 1060 (30.6%) | 6.7% (4.6%-8.8%) | 1.4 (1.2-1.5) | 1.48 (1.34-1.63) * | <0.001 |
| - Shade use | 1625 (38.3%) | 1303 (37.6%) | 0.7% (-1.5%-2.9%) | 1.0 (0.9-1.1) | 1.11 (1.01-1.22) * | 0.03 |
| - Sunglasses use | 3040 (71.6%) | 2449 (70.7%) | 1.0% (-1.1%-3.0%) | 1.0 (0.9-1.2) | 1.07 (0.97-1.18) | 0.21 |
| **Sunscreen type** | | | | | | |
| - Broad-spectrum | 3009 (70.9%) | 2140 (61.7%) | 9.2% (7.0%-11.3%) | 1.5 (1.4-1.7) | 1.63 (0.95-2.81) | 0.08 |
| - SPF >30 | 3560 (83.9%) | 2580 (74.4%) | 9.4% (7.6%-11.3%) | 1.8 (1.6-2.0) | 1.33 (1.14-1.54) * | <0.001 |
| **Skin check** | 3565 (84.0%) | 2874 (82.9%) | 1.1% (-0.6%-2.8%) | 1.1 (1.0-1.2) | 1.15 (1.02-1.31) * | 0.02 |
| **Reaction** | | | | | | |
| - Family doctor visit | 1578 (37.2%) | 1450 (41.8%) | -4.7% (-6.8%- -2.5%) | 0.8 (0.7-0.9) | 0.86 (0.78-0.94) * | 0.002 |
| - Check by friend/family member | 2046 (48.2%) | 1551 (44.8%) | 3.5% (1.2%-5.7%) | 1.1 (1.0-1.3) | 1.09 (0.99-1.19) | 0.08 |
| - Ignore | 178 (4.2%) | 151 (4.4%) | -0.2% (-1.1%- 0.7%) | 1.0 (0.8-1.2) | 0.92 (0.74-1.15) | 0.47 |
| **Worry if mole** | | | | | | |
| - is irregular in shape | 4125 (97.2%) | 3307 (95.4%) | 1.8% (0.9%-2.6%) | 1.7 (1.3-2.1) | 2.34 (1.58-3.44) * | <0.001 |
| - changes colour | 4157 (98.0%) | 3375 (97.4%) | 0.6% (-0.1%-1.5%) | 1.3 (0.9-1.8) | 1.66 (1.03-2.65) * | 0.04 |
| - grew in size | 4150 (97.8%) | 3379 (97.5%) | 0.3% (-0.4%-1.0%) | 1.1 (0.8-1.5) | 1.13 (0.75-1.70) | 0.57 |

*Statistically significant
**Percent difference between individuals with a university education and those without
+Gender and age-adjusted odds ratios

## TABLE S8: DIFFERENCE BETWEEN LGBTQ+ AND NON-LGBTQ+.

| Variable (n,%) | LGBTQ+ N= 226 | NON-LGBTQ+ N= 7537 | Difference (95% CI, p-value) | Odds Ratio (95% CI) | Adjusted Odds Ratio (95% CI) | P-value of Adjusted Odds Ratio |
|---|---|---|---|---|---|---|
| **Personal history of skin cancer** | 27 (12.0%) | 992 (13.2%) | -1.2% (-5.5%-3.1%) | 0.9 (0.6-1.4) | 1.11 (0.73-1.69) | 0.62 |
| - Melanoma | 11 (40.7%) | 287 (28.9%) | 11.8% (-6.9%-30.6%) | 1.7 (0.7-3.9) | | |
| - Squamous Cell Carcinoma | 5 (18.5%) | 122 (12.3%) | 6.2% (-8.6%-2.1%) | 1.6 (0.5-4.5) | | |
| - Basal Cell Carcinoma | 9 (33.3%) | 477 (48.1%) | -14.8% (-32.8%-3.3%) | 0.5 (0.2-1.3) | | |
| **Family history of skin cancer** | 68 (30.1%) | 2453 (32.6%) | -2.5% (-8.5%-3.6%) | 0.9 (0.7-1.2) | 0.93 (0.69-1.25) | 0.62 |
| **Lifetime sunburns (more than 10)** | 154 (68.1%) | 4945 (65.6%) | 2.5% (-3.6%-8.7%) | 1.1 (0.8-1.5) | 0.90 (0.66-1.22) | 0.49 |
| **Lifetime blistering sunburns (more than 1)** | 157 (69.5%) | 5348 (71.0%) | -1.5% (-7.6%-4.6%) | 0.9 (0.7-1.3) | 0.84 (0.62-1.14) | 0.26 |
| **Tanning bed use (more than 1)** | 100 (44.3%) | 3579 (47.5%) | -3.2% (-9.8%-3.3%) | 0.9 (0.7-1.2) | 0.75 (0.56-1.00) | 0.05 |
| **Sun exposure (high-very high)** | | | | | | |
| - Total sun exposure | 49 (21.7%) | 1544 (20.5%) | 1.2% (-4.3%-6.6%) | 1.1 (0.8-1.5) | 1.03 (0.74-1.42) | 0.87 |
| - Recreational sun exposure | 53 (23.5%) | 1704 (22.6%) | 0.8% (-4.7%-6.4%) | 1.0 (0.8-1.4) | 0.97 (0.71-1.33) | 0.84 |
| - Occupational sun exposure | 15 (6.6%) | 258 (3.4%) | 3.2% (-0.1%-6.5%) | 2.0 (1.1-3.4) | 1.67 (0.96-2.90) | 0.07 |
| **Tan in the last 12 months** | 152 (67.3%) | 5599 (74.3%) | -7.0% (-13.2%- -0.8%) | 0.7 (0.5-1.0) | 0.63 (0.47-0.84) * | 0.002 |
| **Sun protection (always)** | | | | | | |
| - Sunscreen use | 87 (38.5%) | 3521 (46.7%) | -8.2% (-14.7%- -1.8%) | 0.7 (0.5-0.9) | 0.70 (0.53-0.93) * | 0.01 |
| - Long sleeve use | 155 (68.6%) | 4684 (62.2%) | 6.4% (0.3%- 12.6%) | 1.3 (1.0-1.8) | 1.32 (0.98-1.79) | 0.07 |
| - Hat use | 86 (38.1%) | 2563 (34.0%) | 4.0% (-2.4%-10.5%) | 1.2 (0.9-1.6) | 1.36 (1.03-1.81) * | 0.03 |
| - Shade use | 82 (36.3%) | 2859 (37.9%) | -1.6% (-8.0%-4.7%) | 0.9 (0.7-1.2) | 1.09 (0.83-1.45) | 0.53 |
| - Sunglasses use | 134 (59.3%) | 5377 (71.3%) | -12.0% (-18.5%- -5.6%) | 0.6 (0.4-0.8) | 0.62 (0.47-0.82) * | <0.001 |
| **Sunscreen type** | | | | | | |
| - Broad-spectrum | 149 (65.9%) | 5017 (66.6%) | -0.6% (-6.9%-5.6%) | 1.0 (0.7-1.3) | 1.36 (0.19-9.95) | 0.76 |
| - SPF >30 | 193 (85.4%) | 5986 (79.4%) | 6.0% (1.3%-10.7%) | 1.5 (1.0-2.3) | 1.90 (1.06-3.43) * | 0.03 |
| **Skin check** | 177 (78.3%) | 6303 (83.6%) | -5.3% (-10.7%- 0.0%) | 0.7 (0.5-1.0) | 0.87 (0.62-1.22) | 0.41 |
| **Reaction** | | | | | | |
| - Family doctor visit | 77 (34.1%) | 2973 (39.5%) | -5.4% (-11.7%- 0.9%) | 0.8 (0.6-1.1) | 0.92 (0.69-1.22) | 0.56 |
| - Check by friend/family member | 121 (53.5%) | 3502 (46.5%) | 7.1% (0.5%-13.7%) | 1.3 (1.0-1.7) | 1.15 (0.88-1.51) | 0.31 |
| - Ignore | 8 (3.5%) | 323 (4.3%) | -0.7% (-3.2%-1.7%) | 0.8 (0.3-1.7) | 0.70 (0.34-1.45) | 0.34 |
| **Worry if mole** | | | | | | |
| - is irregular in shape | 219 (96.9%) | 7261 (96.3%) | 0.6% (-1.7%-2.9%) | 1.2 (0.6-3.0) | 4.73 (0.65-34.28) | 0.12 |
| - changes colour | 223 (98.7%) | 7359 (97.6%) | 1.0% (-0.5%-2.6%) | 1.8 (0.6-8.9) | 1.8 (0.6-8.9) | 0.59 |
| - grew in size | 219 (96.9%) | 7366 (97.7%) | -0.8% (-3.1%-1.5%) | 0.7 (0.3-1.9) | 0.73 (0.26-2.03) | 0.55 |

*Statistically significant
**Percent difference between LGBTQ+ and non-LGBTQ+ communities
+Gender and age-adjusted odds ratios

## TABLE S9: DIFFERENCE BETWEEN MEN AND WOMEN.

| Variable (n,%) | Women N=5648 | Men N= 2202 | Difference (95% CI, p-value) | Odds Ratio (95% CI) | Adjusted Odds Ratio (95% CI) | P-value of Adjusted Odds Ratio |
|---|---|---|---|---|---|---|
| **Personal history of skin cancer** | 667 (11.8%) | 368 (16.7%) | -4.9% (-6.7%- -3.1%) | 0.7 (0.6-0.8) | 0.78 (0.68-0.90) * | <0.001 |
| - Melanoma | 185 (27.7%) | 116 (31.5%) | -3.8% (-9.6%-2.1%) | 0.8 (0.6-1.1) | | |
| - Squamous Cell Carcinoma | 84 (12.6%) | 46 (12.5%) | 0.1% (-4.1%-4.3%) | 1.0 (0.7-1.5) | | |
| - Basal Cell Carcinoma | 336 (50.4%) | 157 (42.7%) | 7.7% (1.4%-14.0%) | 1.4 (1.0-1.8) | | |
| **Family history of skin cancer** | 1942 (34.4%) | 603 (27.4%) | 7.0% (4.8%-9.2%) | 1.4 (1.2-1.6) | 1.33 (1.19-1.49) * | <0.001 |
| **Lifetime sunburns (more than 10)** | 3614 (64.0%) | 1534 (69.7%) | -5.7% (-8.0%- -3.4%) | 0.8 (0.7-0.9) | 0.65 (0.58-0.73) * | <0.001 |
| **Lifetime blistering sunburns (more than 1)** | 4039 (71.5%) | 1527 (69.4%) | 2.2% (-0.1%-4.4%) | 1.1 (1.0-1.2) | 1.08 (0.97-1.22) | 0.17 |
| **Tanning bed use (more than 1)** | 3056 (54.1%) | 657 (29.8%) | 24.3% (22.0%-26.6%) | 2.8 (2.5-3.1) | 2.50 (2.25-2.79) * | <0.001 |
| **Sun exposure (high-very high)** | | | | | | |
| - Total sun exposure | 1120 (19.8%) | 489 (22.1%) | -2.4% (-4.4%- -0.4%) | 0.9 (0.8-1.0) | 0.86 (0.76-0.97) * | 0.01 |
| - Recreational sun exposure | 1153 (20.4%) | 623 (28.3%) | -7.9% (-10.0%- -5.7%) | 0.7 (0.6-0.7) | 0.63 (0.56-0.71) * | <0.001 |
| - Occupational sun exposure | 104 (1.8%) | 174 (7.9%) | -6.1% (-7.2%- -4.9%) | 0.2 (0.2-0.3) | 0.21 (0.16-0.27) * | <0.001 |
| **Tan in the last 12 months** | 4132 (73.2%) | 1679 (76.3%) | -3.1% (-5.2%- -1.0%) | 0.8 (0.8-1.0) | 0.77 (0.69-0.87) * | <0.001 |
| **Sun protection (always)** | | | | | | |
| - Sunscreen use | 2904 (51.4%) | 740 (33.6%) | 17.8% (15.4%-20.2%) | 2.1 (1.9-2.3) | 2.01 (1.81-2.23) * | <0.001 |
| - Long sleeve use | 3086 (54.6%) | 1812 (82.3%) | -27.7% (-29.7%- -25.6%) | 0.3 (0.2-0.3) | 0.27 (0.24-0.31) * | <0.001 |
| - Hat use | 1728 (30.6%) | 962 (43.7%) | -13.1% (-15.5%- -10.7%) | 0.6 (0.5-0.6) | 0.63 (0.57-0.70) * | <0.001 |
| - Shade use | 2247 (39.8%) | 729 (33.1%) | 6.7% (4.3%-9.0%) | 1.3 (1.2-1.5) | 1.47 (1.32-1.64) * | <0.001 |
| - Sunglasses use | 4190 (74.2%) | 1386 (62.9%) | 11.2% (8.9%- 13.6%) | 1.7 (1.5-1.9) | 1.70 (1.53-1.89) * | <0.001 |
| **Sunscreen type** | | | | | | |
| - Broad-spectrum | 3888 (68.8%) | 1336 (60.7%) | 8.2% (5.8%-10.5%) | 1.4 (1.3-1.6) | 0.76 (0.40-1.46) | 0.42 |
| - SPF >30 | 4590 (81.3%) | 1652 (75.0%) | 6.2% (4.2%-8.3%) | 1.4 (1.3-1.6) | 0.85 (0.71-1.01) | 0.07 |
| **Skin check** | 4822 (85.4%) | 1735 (78.8%) | 6.6% (4.6%-8.5%) | 1.6 (1.4-1.8) | 1.70 (1.49-1.93) * | <0.001 |
| **Reaction** | | | | | | |
| - Family doctor visit | 2371 (42.0%) | 723 (32.8%) | 9.1% (6.8%-11.5%) | 1.5 (1.3-1.6) | 1.68 (1.51-1.87) * | <0.001 |
| - Check by friend/family member | 2413 (42.7%) | 1239 (56.3%) | -13.5% (-16.0%- -11.1%) | 0.6 (0.5-0.6) | 0.56 (0.51-0.62) * | <0.001 |
| - Ignore | 221 (3.9%) | 113 (5.1%) | -1.2% (-2.3%- -0.2%) | 0.8 (0.6-1.0) | 0.73 (0.58-0.93) * | 0.009 |
| **Worry if mole** | | | | | | |
| - is irregular in shape | 5517 (97.7%) | 2042 (92.7%) | 4.9% (3.8%-6.1%) | 3.3 (2.6-4.2) | 3.76 (2.61-5.42) * | <0.001 |
| - changes colour | 5554 (98.3%) | 2110 (95.8%) | 2.5% (1.6%- 3.4%) | 2.6 (1.9-3.5) | 2.37 (1.50-3.75) * | <0.001 |
| - grew in size | 5532 (98.0%) | 2133 (96.9%) | 1.1% (0.3%-1.9%) | 1.5 (1.1-2.1) | 1.30 (0.84-2.01) | 0.24 |

*Statistically significant
**Percent difference between men and women
+Gender and age-adjusted odds ratios
